# The claustrum of the bottlenose dolphin *Tursiops truncatus* (Montagu 1821)

**DOI:** 10.3389/fnsys.2014.00042

**Published:** 2014-03-28

**Authors:** Bruno Cozzi, Giulia Roncon, Alberto Granato, Maristella Giurisato, Maura Castagna, Antonella Peruffo, Mattia Panin, Cristina Ballarin, Stefano Montelli, Andrea Pirone

**Affiliations:** ^1^Department of Comparative Biomedicine and Food Science, University of PadovaLegnaro, Italy; ^2^Department of Psychology, Catholic UniversityMilan, Italy; ^3^Department of Translational Research on New Technologies in Medicine and Surgery, University of PisaPisa, Italy; ^4^Department of Veterinary Sciences, University of PisaPisa, Italy

**Keywords:** claustrum, bottlenose dolphin, calcium binding proteins, NPY, somatostatin, insular cortex

## Abstract

The mammalian claustrum is involved in processing sensory information from the environment. The claustrum is reciprocally connected to the visual cortex and these projections, at least in carnivores, display a clear retinotopic distribution. The visual cortex of dolphins occupies a position strikingly different from that of land mammals. Whether the reshaping of the functional areas of the cortex of cetaceans involves also modifications of the claustral projections remains hitherto unanswered. The present topographic and immunohistochemical study is based on the brains of eight bottlenose dolphins and a wide array of antisera against: calcium-binding proteins (CBPs) parvalbumin (PV), calretinin (CR), and calbindin (CB); somatostatin (SOM); neuropeptide Y (NPY); and the potential claustral marker Gng2. Our observations confirmed the general topography of the mammalian claustrum also in the bottlenose dolphin, although (a) the reduction of the piriform lobe modifies the ventral relationships of the claustrum with the cortex, and (b) the rotation of the telencephalon along the transverse axis, accompanied by the reduction of the antero-posterior length of the brain, apparently moves the claustrum more rostrally. We observed a strong presence of CR-immunoreactive (-ir) neurons and fibers, a diffuse but weak expression of CB-ir elements and virtually no PV immunostaining. This latter finding agrees with studies that report that PV-ir elements are rare in the visual cortex of the same species. NPY- and somatostatin-containing neurons were evident, while the potential claustral markers Gng2 was not identified in the sections, but no explanation for its absence is currently available. Although no data are available on the projections to and from the claustrum in cetaceans, our results suggest that its neurochemical organization is compatible with the presence of noteworthy cortical inputs and outputs and a persistent role in the general processing of the relative information.

## Introduction

Dolphins (Delphinidae) are carnivore marine mammals belonging to the Infraorder Odontoceti (toothed whales). Together with the Infraorder Mysticeti (baleen whales) toothed whales belong to the taxon Cetacea which is today grouped together with Artiodactyla (even-toed hooved mammals) into the single order Cetartiodactyla (Geisler and Uhen, 2005; Thewissen et al., 2007). This relationship may explain some of their anatomical conformities and common evolutionary adaptations with domestic animals like the cow and the pig (for general reference see Slijper, 1979). However the brain of dolphins possesses some strikingly unique features, including compression of the longitudinal axis and expansion of the temporal width, pronounced rotation along the transverse (inter-insular) axis, essential absence of the olfactory lobe and nerves, intense folding of the cerebral cortex accompanied by reduced thickness, and general uniformity of columnar organization in the different topographical areas. On the other hand, the virtual absence of a typical layer IV in cetaceans (for a comprehensive review see Manger, 2006) is common also to other Cetartiodactyla and Ungulates in general (Hof et al., 1999, 2000). Physiological studies on brain functions and internal connections are obviously restricted by ethical reasons and by the consequent limited number of published studies. However, a review of the available information suggests that functional localizations differ from terrestrial mammals (Oelschläger and Oelschläger, 2009). The visual cortex is not located in the occipital pole, but shifted dorsally and placed longitudinally, separated from the inter-hemispheric scissure by the peculiar paralimbic lobe, and accompanied laterally by the elongated acoustic cortex (Lende and Akdikmen, 1968; Kesarev and Malofeeva, 1969; for review see Morgane et al., 1986). Motor and somatosensory cortices are pushed rostrally almost entirely on the frontal aspect of the brain, in relation also to the particularly advanced position of the cruciate sulcus (Lende and Akdikmen, 1968; Kesarev and Malofeeva, 1969).

The claustrum is considered to be reciprocally connected to several cortical areas, and to possess direct involvement in the processing of sensorimotor information (Crick and Koch, 2005), with a special relationship to the visual cortex in the cat (Olson and Graybiel, 1980; Minciacchi et al., 1995) and monkey (Remedios et al., 2010). The topographic shift of the dolphin visual cortex to a location parallel to the inter-hemispheric cleft, and the profound modifications of several modalities of somatosensory inputs (i.e., related to the virtual absence of taste buds in the tongue, reduction of the hand, and disappearance of the hind limb) suggest possible functional adaptative modifications of the claustrum. The claustrum of dolphins (and cetaceans in general) has never been specifically described, although references to its position and relationship with the complex insular cortex and pocket (Jacobs et al., 1984) indicate a predominance of the infrainsular part, corresponding to the central core of the insular cortex in parasagittal section. In fact the same authors report that in the posterior and dorsal insular regions of the bottlenose dolphin (*Tursiops truncatus*), the claustrum is “either absent or present as a discontinuous cell band beneath the insular cortex”, and in their study the diagrams show a close proximity, if not contiguity, of the ventral claustrum with the sylvian cleft in the more caudal extension (Jacobs et al., 1984). The contiguity of the cetacean claustrum with the cerebral cortex was noted by other authors in the harbor porpoise (Jelgersma, 1934) and reported in a comprehensive review (Jansen and Jansen, 1969).

In the present study we intend to investigate the topography and selected neurochemical characteristics of the claustrum in the bottlenose dolphin, the most widely studied member of the Family Delphinidae. The neurochemical organization of the claustrum, as defined by the expression of calcium-binding proteins (CBPs), selected modulators (neuropeptide Y, NPY; somatostatin, SOM), and the recognized claustral marker Gng2 (Mathur et al., 2009) may help understand whether this structure maintains in dolphins the organization now considered typical of terrestrial mammals, and whether its neurochemical characteristics are similar to those of other mammalian models.

## Materials and methods

### Tissue samples

In this study we used samples of the claustrum obtained from the brains of eight bottlenose dolphins (see Table 1) stored in the *Mediterranean marine mammal tissue bank* (MMMTB) of the University of Padova at Legnaro, Italy. The MMMTB is a CITES recognized (IT020) research center and tissue bank (Ballarin et al., 2005), sponsored by the Italian Ministry of the Environment and the University of Padova, with the aim of harvesting tissues from wild and captive cetaceans and distributing them to qualified research centers worldwide.

**Table 1:** **Details of the sampled bottlenose dolphins**.

**Specimen**	**Sex**	**Origin**	**Age (years)/length/weight**	**Formalin**	**Frozen**
ID # 95	F	wild	11 (pregnant adult)/285 cm		x
ID # 107	M	captive	9 /250 cm	x	x
ID # 110	M	wild	> 2/190 cm /74 kg		x
ID # 114	M	captive	newborn/115 cm	x	
ID # 133	F	captive	adult (age uncertain) /248 cm	x	
ID # 139	M	captive	12 /268 cm/198 kg	x	x
ID # 146	M	captive	3.5/226 cm	x	
ID # 159	M	captive	> 40 /328 cm	x	

**Figure 1:**
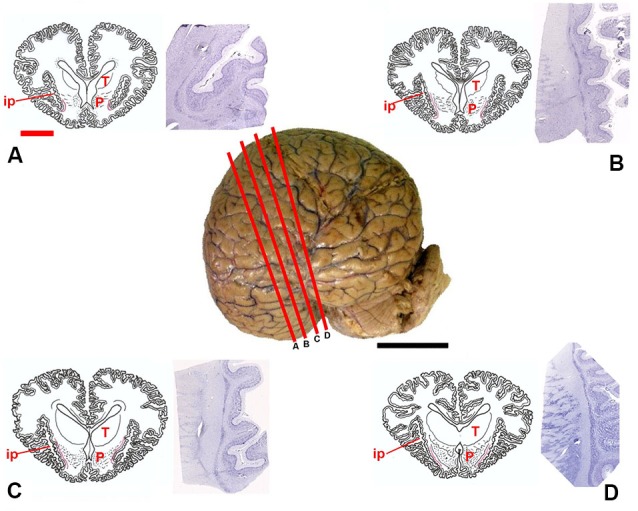
**Position of the claustrum in *Tursiops truncatus***. Center, lateral photograph of the adult brain; the orange bars **(A–D)** approximate the levels of subsequent sections. The drawings **(A–D)** are based on composite renderings of coronal sections of the dolphin brain, sided by Nissl preparations obtained approximately at the corresponding levels. In the drawings the claustrum is represented by a thin red line. Note the reduction of the endopiriform cortex ventral to the basal ganglia; P, putamen; T, thalamus; ip, insular pocket. Black scale bar (lateral photograph of the dolphin brain) = 5 cm; orange scale bar (drawings) = 2 cm.

Tissue samples consisted of blocks approximately 1 cm thick, including the claustrum, surrounded by portions of the adjoining structures (extreme and external capsules, insular cortex, putamen), carefully dissected (Figure 1) during post-mortem procedures performed in the necropsy room of the Department of Comparative Biomedicine and Food Science of the University of Padova at Legnaro. Post-mortem delay before actual sampling varied between 18 and 40 h. The samples were fixed by immersion in 4% buffered formalin, washed in phosphate saline buffer (PBS) 0.1 M, pH 7.4 and subsequently either processed for paraffin embedding or frozen by immersion in liquid nitrogen-chilled isopentane at −30°C. Sections were cut either with a microtome (6 μm) or a cryostat (20 μm) and subsequently processed for immunohistochemistry (see below). For each tissue block, one section out of ten was treated for Nissl stain for general outline, reference and topography.

### Immunohistochemistry

A rabbit polyclonal anti-calretinin (CR) antibody (sc-50453; Santa Cruz Biotech., Inc., Santa Cruz, CA; dilution 1:200), a mouse monoclonal anti-parvalbumin (PV) antibody (Clone PA-235, Cat. # P-3171; Sigma-Aldrich, St. Louis, MO, USA; dilution 1:3000), a mouse monoclonal anti-calbindin-D-28K (CB) antibody (Clone CB-955, Cat. # C9848, Sigma-Aldrich, St. Louis, MO, USA; dilution 1:3000), a rabbit polyclonal anti-CB-D-28K antibody (Cat. # CB38A, Swant, Bellinzona, Switzerland; diluition 1:10000), a rabbit polyclonal anti-NPY antibody (ab30914; abcam; dilution 1:3000), a rabbit polyclonal anti-Gng2 antibody (HPA003534; Sigma-Aldrich, dilution 1:100), and a rabbit polyclonal anti-SOM antibody (ab103790; abcam; dilution 1:700) were used in this study. Epitope retrieval was carried out at 120°C in a pressure cooker for 5 min using a Tris/EDTA buffer pH 9.0. Sections were rinsed in PBS and incubated in 1% H_2_O_2_-PBS for 10 min, then pre-incubated in PBS with 0.3% Triton X-100 (TX) (Sigma-Aldrich, St. Louis, MO, USA) and 5% normal goat serum (Vector Labs, Burlingame, CA) to reduce non-specific staining. Sections were subsequently incubated overnight in a humid chamber at 4°C with the primary antibody diluted in PBS with 0.3% TX and 1% normal goat serum. After several washings in PBS, sections were incubated for 1 h at room temperature with biotinylated goat anti-rabbit (for CR, NPY and SOM) or biotinylated goat anti-mouse secondary antibodies (for PV and CB) (Vector Labs, Burlingame, CA), diluted 1:300 in PBS. Sections were then washed for 3 × 10 min in PBS, and incubated for 1 h at room temperature in avidinbiotin-horseradish peroxidase complex (PK-6100; Vector Labs, Burlingame, CA). After washing for 3 × 10 min in Tris/HCl (pH 7.6), peroxidase activity was detected by incubation in a solution of 0.125 mg/ml diaminobenzidine (Sigma-Aldrich, St. Louis, MO, USA) and 0.1% H_2_O_2_ in the same buffer for 10 min or by a VIP substrate Kit for peroxidase (Cat. # SK-4600, Vector Labs, Burlingame, CA).

The amino acid sequence of the proteins investigated in this article in the claustrum of *Tursiops truncatus* were compared with those of other mammals (and especially the rat). For this aim we used the Ensembl genomic database.^1^ The sequence of NPY, SOM, CB and CR is shared for over 93%, whereas correspondence for Gng2 and PV is over 70%. The specificity of the immunohistochemical staining was tested in repeated trials as follows: substitution of either the primary antibody, the anti-rabbit or anti-mouse IgG, or the ABC complex by PBS or non-immune serum. Under these conditions the staining was abolished.

## Results

### General topography and shape

Based on the examinations of macroscopic slices of the brain, followed by analysis of Nissl-stained sections, the claustrum was detected in all the examined specimens, including newborns. The topography of the claustrum observed in our experimental series reflects the general orientation of the dolphin brain, in which the lateral (temporal) lobes grow considerably, and the lower (partially olfactory) divisions of the telencephalon are reduced. The position of the claustrum was clearly identified lateral to the conspicuous putamen and medial to the insular formation and relative insular pocket (Figure 1).

In Nissl-stained sections the claustrum appeared thin (not thicker than 1–2 mm) and dorso-ventrally elongated (up to 3.5 cm), without an evident endopiriform root as commonly found in terrestrial mammals. The ventralmost limits of the claustrum apparently touch the insular cortex with virtual disappearance of the *capsula extrema*.

### Immunohistochemical data

#### Calretinin

**Figure 2:**
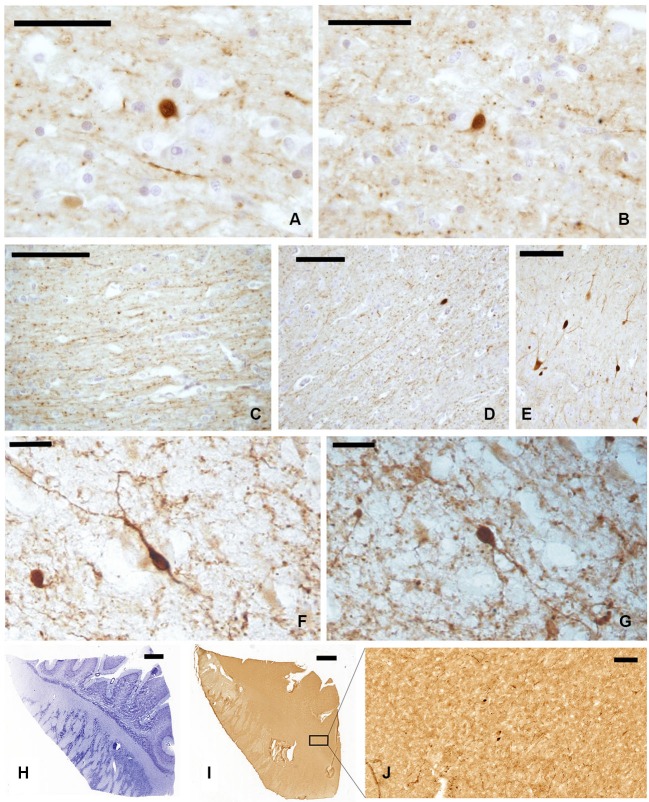
**CR-ir neurons and fibers in the claustrum**. **(A–D)** Images derive from paraffin-embedded sections. **(A, B)** Small unipolar neurons surrounded by beaded fibers; **(C, D)** several CR-ir beaded fibers cross the whole length of the claustrum. **(E)** CR-ir neurons in the insular cortex. **(F, G)** Immunostained neurons in frozen sections; **(H–J)** localization of CR-ir neurons in whole sections (**(H)** Nissl stain, **(I–J)** immunocytochemistry); **J** represent an enlargement of the black rectangle in **I** Scale bars: **A, B** = 50 μm; **C, D, E, J** = 100 μm; **F, G** = 20 μm; **H, I** = 2 mm.

In our experimental series, we observed a strong presence of CR-immunoreactive (-ir) neurons and fibers, both in paraffin-embedded (Figures 2A–E) and frozen sections (Figures 2F, G). Positive cells appeared as mono- and bi-polar small (approx. 10 μm) neurons, with a round or fusiform soma. The morphology of CR-ir neurons in the claustrum was very different from that of the typical CR-ir neurons in the cortical columns of the adjacent insula (Figure 2E).

The distribution of CR-ir elements (Figures 2H–J) did not show any specific segregation and the neurons were diffuse in all the parts of the claustrum, although immunostained cells were scarcer at its dorsal and ventral extremities.

#### Calbindin

**Figure 3:**
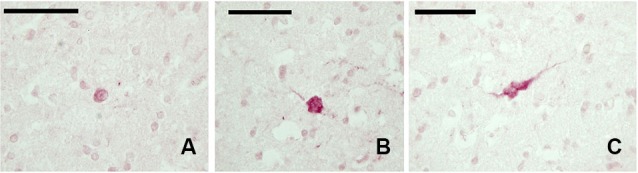
**CB-ir neurons in the claustrum.** **(A–C)** Immunoreactive neurons in paraffin-embedded sections. Scale bars etc.

The examined sections showed a diffuse but weak expression of CB-ir elements (Figures 3A–C). The few positive elements displayed a small (approx. 10 μm) mono- or bipolar soma. Positive fibers were evident throughout the claustrum.

#### Parvalbumin

The immunostaining for PV revealed no positive cell or fiber in the claustrum of all the animals examined.

#### Neuropeptide Y

**Figure 4:**
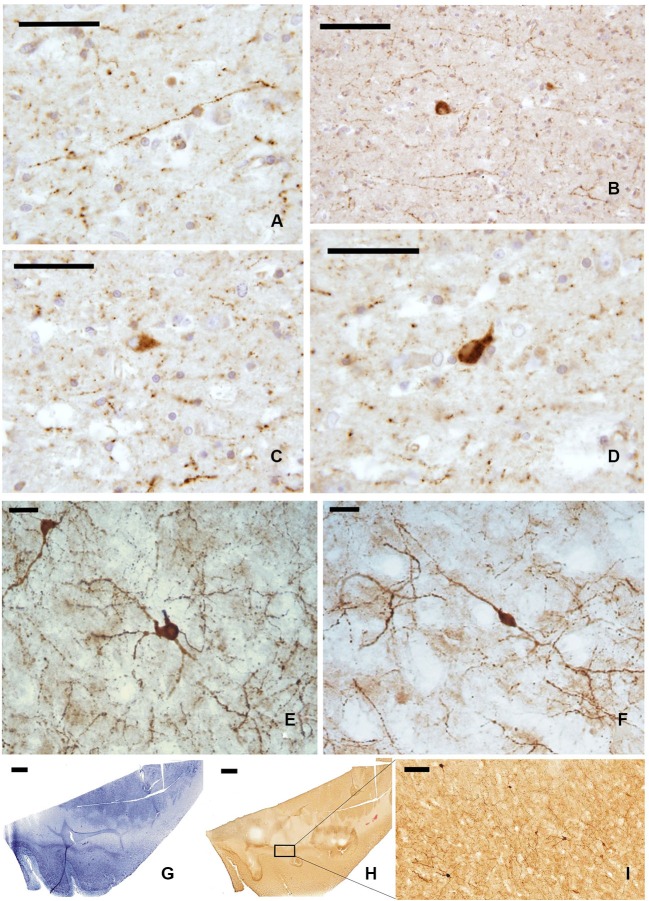
**NPY-ir fibers **(A, B)** and neurons **(C, D)** in paraffin-embedded sections of the claustrum; **(E, F)** NPY positive neurons in frozen sections; **(G–I)** localization of NPY-ir neurons in whole sections (**(G)** Nissl stain, **(H–I)** immunocytochemistry); **I** represent an enlargement of the black rectangle in **H****. Scale bars = **A, C, D** = 50 μm; **B, I** = 100 μm; **E, F** = 20 μm;. **G, H** = 2 mm.

The whole length of the claustrum is crossed by a mesh of NPY-ir beaded nerve fibers (Figures 4A, B), with some unipolar, pseudo-unipolar, and multipolar small-medium (15–20 μm) neurons (Figures 4C, D). In cryostat cut sections some of these neurons displayed a well-ramified dendrite arborization (Figures 4E, F). Also the distribution of NPY-ir elements (Figures 4G–I) did not show any specific segregation, although immunostained cells were scarcer at the dorsal and ventral extremities of the claustrum.

#### Somatostatin

**Figure 5:**
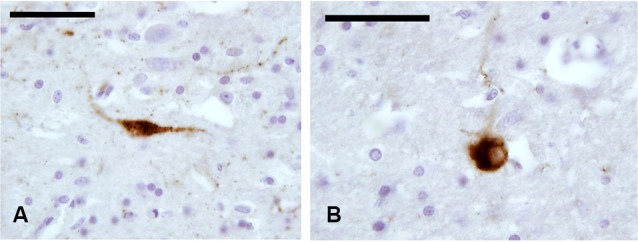
**SOM-ir fibers and neurons in the claustrum**. **(A–B)** Images from paraffin-embedded sections. Scale bars etc.

In our experimental series, we identified a few SOM-ir neurons in the claustrum (Figure 5). Immunostained elements appeared either as slender bipolar neurons or spherical, with medium dimensions (approx. 20–25 μm). Fibers were rare.

#### Gng2

In our experimental series the claustral marker Gng2 was not identified in any cell or fiber of the claustrum or adjacent brains structures.

## Discussion

**Figure 6:**
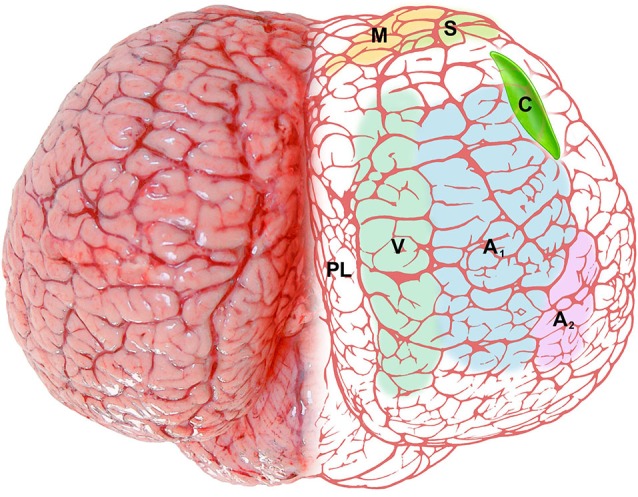
**Schematic representation of the claustrum (C, bright green) of the bottlenose dolphin shown through the dorsal aspect of the brain**. The pale colors on the right side represent functional areas of the cortex: M (yellow), motor cortex; S (green), somatosensory cortex; V (gray-green), visual cortex; A_1_ (pale blue), primary auditory cortex; A_2_ (pale pink), secondary auditory cortex; PL (no color), paralimbic lobe.

The position and relationships of the claustrum in the bottlenose dolphin described here is based on the analysis of macroscopic brain slices and Nissl stained sections. The location of the claustrum (Figure 6) reflects the changes in the general outline of the brain and consequently the modifications of its internal organization and topography. The absence of a complete endopiriform cortex as such (due to the lack of olfactory bulbs and related structures) limits the development of the so-called “endopiriform” part of the claustrum, and modifies its ventral outline. The rostro-ventral part of the dolphin claustrum surrounds the ventral borders of the insular pocket, but shows no medial projections towards the midsagittal plane as in terrestrial mammals including man. The extreme reduction of the Ammon’s horn and the hippocampal formation in general (Morgane et al., 1982), sensibly changes the disposition of ventro-lateral structures in the temporal lobe, and their reciprocal relationships. In man and other primates the caudalmost part of the claustrum terminates dorsal to the tail of the caudate nucleus and the hippocampus. This latter disposition is absent in the dolphin, in which the pronounced rotation of cerebral components along the transverse (inter-insular) axis (Figure 1; for detailed description and an interpretation see Morgane et al., 1980), and the reduction of olfaction-related limbic structures places the caudal extremity of the claustrum more anteriorly (see Figure 6). In a dated but well known review on the cetacean nervous system (Jansen and Jansen, 1969), it was noted that “the claustrum extends rostrolaterally beyond the limits of the putamen”, with reference to the harbor porpoise (Jelgersma, 1934). The disposition of the claustrum that we describe here in *Tursiops truncatus* overlaps what reported for the common dolphin *Delphinus delphis*, at least based on a series of transverse sections of the brain (Pilleri et al., 1980).

Our data confirm what was reported in the drawings of the seminal paper on the bottlenose dolphin insula by Jacobs et al. (1984), in which the ventral extremity of the claustrum is attached to the insular cortex and the opercular gyri at the level of the insular pocket. We also noted contiguity between the insular cortex and the ventral extremity of the claustrum. We emphasize this topographical relationship in view of its ontogenetic significance for the origin of the claustrum. As well known, the claustrum may derive from (a) the putamen and basal ganglia; (b) the insular cortex; or possibly (c) a combination of both (Edelstein and Denaro, 2004). A recent study (Pirone et al., 2012), performed on post-mortem human brains, identified the presence of a potential claustral marker, the protein Gng2, formerly developed in rodents (Mathur et al., 2009), in the insular cortex and claustrum, but not in the putamen, thus suggesting a possible common origin of the former two structures. There is presently no information on how much the structure of Gng2 is conservative among mammals, and the eventual presence of different isoforms of this protein in cetaceans may explain the lack of immunoreactivity in our experimental series. However, contiguity between cortex and claustrum, as we observed in the bottlenose dolphin, may reinforce the insular hypothesis.

The presence of CBPs in the claustrum has been described in several mammalian species (Reynhout and Baizer, 1999; Ángeles Real et al., 2003; Wojcik et al., 2004; Rahman and Baizer, 2007), and a recent detailed study reports the distribution of PV in the human claustrum (Hinova-Palova et al., 2013). Former studies on the distribution of CBPs in the auditory and visual cortex of the bottlenose dolphin (Glezer et al., 1995, 1998) identified only very few PV-ir neurons, contrarily to what observed in the macaque *Macaca fascicularis*, in which PV was present or even prevalent in both systems. Furthermore, PV-ir neurons in the visual cortex of selected cetacean species were scarce except in layers IIIc/V and VI (Glezer et al., 1993). On the other hand, the GABA component seems not different between cetaceans and terrestrial mammals (Garey et al., 1989). Our data indicate that PV is not expressed in the claustrum of the bottlenose dolphin, a fact that may be related to the paucity of PV-ir neurons in the visual cortex of the same species described in the reports cited above. Considering that the PV amino acid sequence in dolphins shows a 70% identity with the corresponding protein of terrestrial mammals, additional studies are required to further clarify this issue and its important implications in terms of claustro-visual projections. However we’d like to emphasize that our experimental procedures were performed under the same conditions applied in our laboratory to the primate claustrum in which a clear, well evident presence of a network of PV-containing elements was observed (data not shown), even when the brains suffered a post-mortem interval prior to sampling possibly longer than that of the bottlenose dolphins described here.

CR-ir neurons were described in the visual cortex of *Tursiops truncatus* (Glezer et al., 1992) and CR- and CB-ir neurons were identified especially in the auditory system of the same species (Reynhout and Baizer, 1999). In our series mono- or bi-polar neurons CR-ir were easily identified in the claustrum, and a few CB-ir neurons were also evident. The dorsal claustrum receives important visual projections from the occipital cortex in the cat (LeVay and Sherk, 1981), while the visual claustrum is instead ventral in primates (Remedios et al., 2010). The apparent reduction of the ventral part of the claustrum that takes place in the bottlenose does not imply curtailed visual projections, but may simply reflect a different topography of visual inputs/outputs as reported in other species. We emphasize here that the dolphin visual cortex shifts dorsally (see Figure 6), and a re-shaping of the internal organization of the claustrum may be plausible, with the visual domains potentially moving dorsally within the nucleus. The visual cortex of the dolphins shows a specific organization, containing both a heterolaminar part/component, with an incipient layer IV, and a homolaminar one, where layer IV is absent, as elsewhere in the cortex (Morgane et al., 1988), a feature typical of Cetartiodactyla (Hof et al., 1999, 2000). The mostly agranular visual cortex of the bottlenose dolphin has been discussed in details (Garey et al., 1985; Morgane et al., 1990), but its projections remain undetected. In a retrograde tracer study performed on *Phocoena phocoena* (Revishchin and Garey, 1990), the visual cortex was found to project to the lateral geniculate nucleus and inferior pulvinar, but no connection was precisely identified outside the thalamus. We note here that PV-ir elements are particularly abundant in layer IV of the somatosensory and auditory cortices of several mammalian orders (Sherwood et al., 2009), but CR and CB are the predominant CBPs in the Cetartiodactyla cortex (Hof et al., 1999, 2000), lacking layer IV. Our data indicate that in the bottlenose dolphin CR is the prevalent CBP in the claustrum, thus suggesting its potential role for reciprocal claustro-cortical connections.

The cellular types that we illustrate here correspond to those described in other species (for review see Edelstein and Denaro, 2004). NPY and SOM are expressed in neurons of the primate (Smith et al., 1985) and rodent claustrum (Kowiański et al., 2008). Due to the plausible common origin of claustrum and insular cortex (Pirone et al., 2012), we can only speculate that claustral and cortical interneurons may play similar functional roles. In the neocortex, Martinotti cells, which target the apical tuft of pyramidal dendrites, express SOM (Markram et al., 2004). Similarly, NPY expressing neurons are able to modulate Ca^2+^-dependent currents in the distal dendrites in pyramidal neurons (Hamilton et al., 2013). On the contrary, PV interneurons mainly target the soma and proximal dendrites of principal neurons (Markram et al., 2004). Therefore, given the presence of NPY and SOM interneurons in the dolphin claustrum, along with the absence of PV, it is possible that the cetacean claustrum might display a prevalence of interneurons with putative synapses onto distal dendrites of projecting neurons. The functional significance of this peculiar feature would deserve further examination.

Our observations confirmed the general outline of the mammalian claustrum also in the bottlenose dolphin, even if the reduction of the piriform lobe modifies the ventral relationships with the cortex.

Although no data are available on the projections to and from the claustrum in cetaceans, our results provide evidence that its neurochemical organization is compatible with the presence of cortical inputs and outputs and a persistent role in the general processing of the relative information. PV-containing interneurons, absent in the claustrum of the bottlenose dolphin, are important fast-spiking elements in the cortex (Moore et al., 2010) and neostriatum (Tepper et al., 2004). Whether the particular display of CBPs in the dolphin claustrum may be functionally related to the structural organization of the cortex (Morgane and Jacobs, 1972; Kern et al., 2011); to the shift in the functional areas (Oelschläger and Oelschläger, 2009); to the virtual absence of binocular vision and the fact that the retina projects almost exclusively to the contralateral hemisphere (Ridgway, 1990; Tarpley et al., 1994); or to the peculiar mono-hemispheric sleep pattern (Mukhametov et al., 1977; see Lyamin et al., 2008 for a recent review), awaits further verification.

## Conflict of interest statement

The authors declare that the research was conducted in the absence of any commercial or financial relationships that could be construed as a potential conflict of interest.

## Footnotes

^1^www.ensembl.org
